# Nanomechanical Study of Enzyme: Coenzyme Complexes: Bipartite Sites in Plastidic Ferredoxin-NADP^+^ Reductase for the Interaction with NADP^+^

**DOI:** 10.3390/antiox11030537

**Published:** 2022-03-11

**Authors:** Sandra Pérez-Domínguez, Silvia Caballero-Mancebo, Carlos Marcuello, Marta Martínez-Júlvez, Milagros Medina, Anabel Lostao

**Affiliations:** 1 Instituto de Nanociencia y Materiales de Aragón (INMA), CSIC-Universidad de Zaragoza, 50009 Zaragoza, Spain; perezdom@uni-bremen.de (S.P.-D.); silvia.caballero@ist.ac.at (S.C.-M.); cmarcuel@unizar.es (C.M.); 2 Laboratorio de Microscopías Avanzadas (LMA), Universidad de Zaragoza, 50018 Zaragoza, Spain; 3 Departamento de Bioquímica y Biología Molecular y Celular, Facultad de Ciencias, Instituto de Biocomputación y Física de Sistemas Complejos (BIFI) (GBsC-CSIC Joint Unit), Universidad de Zaragoza, 50018 Zaragoza, Spain; mmartine@unizar.es; 4 Fundación ARAID, 50018 Zaragoza, Spain

**Keywords:** ferredoxin NADP^+^ reductase, atomic force microscopy, dynamic force spectroscopy, NADP^+^, protein–ligand (substrate) interactions, single-molecule methods, flavoproteins, functionalization, nanomechanics, mechanical stability

## Abstract

Plastidic ferredoxin-NADP^+^ reductase (FNR) transfers two electrons from two ferredoxin or flavodoxin molecules to NADP^+^, generating NADPH. The forces holding the *Anabaena* FNR:NADP^+^ complex were analyzed by dynamic force spectroscopy, using WT FNR and three C-terminal Y303 variants, Y303S, Y303F, and Y303W. FNR was covalently immobilized on mica and NADP^+^ attached to AFM tips. Force–distance curves were collected for different loading rates and specific unbinding forces were analyzed under the Bell–Evans model to obtain the mechanostability parameters associated with the dissociation processes. The WT FNR:NADP^+^ complex presented a higher mechanical stability than that reported for the complexes with protein partners, corroborating the stronger affinity of FNR for NADP^+^. The Y303 mutation induced changes in the FNR:NADP^+^ interaction mechanical stability. NADP^+^ dissociated from WT and Y303W in a single event related to the release of the adenine moiety of the coenzyme. However, two events described the Y303S:NADP^+^ dissociation that was also a more durable complex due to the strong binding of the nicotinamide moiety of NADP^+^ to the catalytic site. Finally, Y303F shows intermediate behavior. Therefore, Y303, reported as crucial for achieving catalytically competent active site geometry, also regulates the concerted dissociation of the bipartite nucleotide moieties of the coenzyme.

## 1. Introduction

In living cells, enzymatic processes are catalyzed by the cooperation of a number of enzymes and coenzymes that, in successive steps, transform metabolites into a variety of products. Furthermore, enzymes are exploited in a variety of manufacturing processes such as the synthesis of medicines [1], food processing, purifying factory effluents, and pollution in water and soils [2], among others. Despite the great power of enzymatic processes, their potential has not been fully exploited, mainly due to the limited understanding of the mechanisms regarding the assembly and dissociation between reacting molecules. Recent advances in imaging methods have demonstrated that it is possible to make direct observations of the dynamic behavior of single molecules [3,4] and to determine the mechanisms of action at the single-molecule level [5,6,7]. Among the available single-molecule methods, those designed to measure forces between molecules or within an individual molecule stand out [8]. The most broadly used technique in this area is atomic force microscopy (AFM) [9] in force spectroscopy mode [10], with other techniques also available, such as biomembrane force probes [11], laminar flow chambers [12], optical tweezers [13], and magnetic tweezers [14]. These approaches allow us to manipulate and measure signals from individual entities and follow their motions at the nanometer scale, as well as to apply and measure forces over a large range, from pico to milli Newtons, depending on the method used. This research area in the dynamic force spectroscopy (DFS) modality tackles the complex relationship between force, lifetime, and chemistry in single molecular bonds, and it is used to analyze the dynamics of the observed process by measuring force as a function of the velocity at which it is applied, termed the loading rate R. The processes that are accessible with this approach range from the stretching of nucleic acids [15], protein unfolding [16], dissociation of ligand–receptor complexes under force [10], and even unraveling enzyme catalysis mechanisms [17], among others. The results given by the use of these techniques have the potential to provide fundamental insights into biological processes, which are critical for a better understanding of molecular dynamics and function.

**Figure 1:**
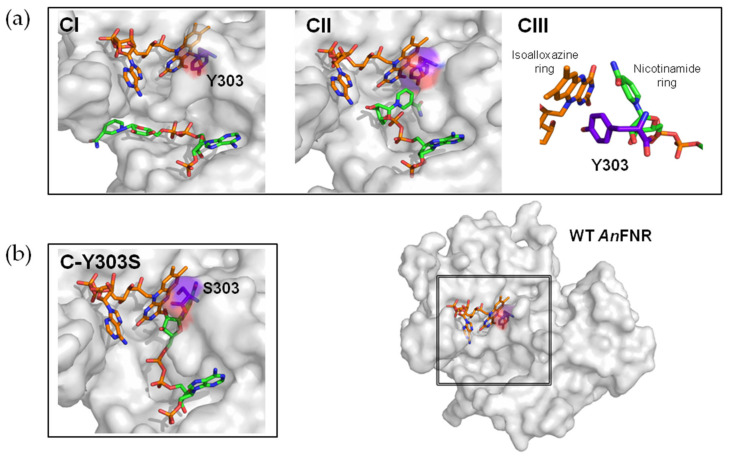
Proposed steps for the interaction of NADP^+^ with AnFNR to attain a catalytically competent complex as depicted by available structural models. (**a**) Detail of the FNR active site in three models of ternary complexes. C-I and C-II are crystallographic models for FNR_ox_:NADP^+^ complex (PDBs 1quf and 1gjr, respectively) related with the initial recognition and binding of the 2-P-AMP moiety, and the subsequent binding of the pyrophosphate moiety of the coenzyme NADP^+^. In none of these structures, the nicotinamide redox moiety of the coenzyme attaches a catalytically competent association to the flavin isoalloxazine ring, because the C-terminal Y303 stacks against the isoalloxazine. C-III shows the organization at the active site of a computationally optimized model that allows stacking of the coenzyme nicotinamide to the isoalloxazine, compatible with HT, and where Tyr303 contributes to nicotinamide allocation [27,30]. (**b**) C-Y303S stands for the crystal structure of the Y303S FNR_ox_:NADP^+^ crystallographic complex (PDB 2bsa), where strong isoalloxazine–nicotinamide stacking prevents the photosynthetic HT [29]. FAD and NADP^+^ are represented in CPK sticks with carbons colored in orange and green, respectively. The C-terminal residue (Y303 or S303) is shown in CPK with carbons in violet purple. The bottom panel on the right shows the corresponding structure of WT AnFNR (PDB 1que), where the studied region in the rest of the panels is highlighted inside a square.

In this study, DFS by AFM was used to analyze the dissociation under the force of ferredoxin–NADP^+^ reductase (FNR) from its nicotinamide adenine dinucleotide phosphate (NADP^+^) substrate at the single-molecule level. During photosynthesis, ferredoxin−NADP^+^ reductase (FNR) catalyzes the electron transfer from ferredoxin (Fd), or flavodoxin (Fld) in case of iron deficiency in the medium, to NADP^+^ via its FAD cofactor to provide reducing power to the energetic metabolism in the form of NADPH [18,19,20]. Indeed, the overall process for NADPH formation involves at least two transient Fd:FNR:NADP^+^ complexes, where the initially preformed FNR_ox_:NADP^+^ complex subsequently binds two Fd (or Fld) molecules successively; the first reduces the enzyme to the semiquinone state, FNR_sq_, and the second reduces the enzyme to the fully reduced hydroquinone state, FNR_hq_. Finally, upon production of the Fd_ox_:FNR_hq_:NADP^+^ organization, hydride transfer (HT) from FNR_hq_ to NADP^+^ takes place [20]. The very first steps in this process include the recognition of the NADP^+^ coenzyme by FNR_ox_. Three FNR sites are relevant to achieve specific and catalytically competent NADP^+^ binding (Figure 1) [21]. The 2′-P-AMP moiety of NADP^+^ is recognized by Ser, Arg, and Tyr residues (Ser223, Arg224, Arg233, and Tyr235 in *Anabaena* FNR (AnFNR)) and this induces conformational changes that facilitate the subsequent binding of the pyrophosphate (accommodated by loops 155–160 and 261–268 in AnFNR) and the nicotinamide mononucleotide (NMN) moieties of the coenzyme [22,23]. Eventually, the efficiency of the HT event depends on the final proper orientation of the N5 of the FAD isoalloxazine ring and the C4 of the coenzyme nicotinamide ring [20]. To achieve such organization, the C-terminal Tyr (Y303 in AnFNR) has to be displaced (Figure 1) [21,24,25]. Site-directed mutagenesis and biocomputational simulations have shown that the side chain of this C-terminal Tyr is key to providing the optimum geometry between reacting rings to provide arrangements compatible with HT (Figure 1) [26,27]. Thus, the replacement of this Tyr by Phe, Trp, or Ser has shown that, despite not being involved in the HT itself, this C-terminal residue modulates the flavin midpoint reduction potential, the NADP^+^⁄H binding affinity, and the selectivity for NADPH, as well as the formation of the catalytically competent complex and release of the NADPH product once the HT reaction takes place [24,28,29].

AFM has been previously used to determine the conformational changes in novel flavoenzyme families upon redox partner binding [31]. Nevertheless, the changes in AnFNR upon NADP^+^ binding are not large enough to be visualized and discerned by AFM imaging. The dissociation under force of FNR complexes with Fd and Fld has been successively analyzed exhaustively by DFS [32,33,34]. The interaction of FNR with Fd rendered longer lifetimes, indicating a much stronger and specific interaction than within the complex formed with Fld. The Fld:FNR complex showed a higher bond probability and two possible dissociation pathways, contrary to the one-event dissociation kinetics observed for the Fd:FNR complex. These results agreed with former functional characterizations and with a more promiscuous recognition process for the Fld:FNR interaction, closer to a dynamic ensemble model, but depicting greater Fd:FNR specificity in the interaction [33,34]. Here, we aim to better understand the very first complex formed in the process, FNR_ox_:NADP^+^, by evaluating its dissociation process under force by DFS. In addition to the WT enzyme, we characterize three different mutants at the C-terminal Tyr of FNR to evaluate its implication. As DFS requires the strong immobilization of molecules of receptors and ligands in a nano-flat substrate and the AFM tip apex, respectively, some functionalization procedures were designed in advance. As far as we know, this is the first enzyme:coenzyme complex analyzed nanomechanically at the single-molecule level, giving a new analytical perspective to the study of enzymes, and providing new methods for the study of other transient complexes that involve coenzyme binding.

## 2. Materials and Methods

### 2.1. Protein Labeling and Immobilization of FNR on Mica

The recombinant WT FNR from *Anabaena* PCC7119 and its Y303S, Y303F, and Y303W variants used in this study were prepared as previously described [29]. FNR molecules in 50 mM Tris/HCl, pH 8.0 were modified with 20 mM sulfosuccinimidyl 6-(3′-[2-pyridyldithio]propionamido) hexanoate (Sulfo-LC-SPDP; Thermo Scientific Pierce, Waltham, MA, USA) to obtain the FNR-PDP tagged proteins, as elsewhere reported [35,36]. FNR–PDP was purified from the excess of non-reacted crosslinker molecules by using Sephadex G-25 size-exclusion PD-MiniTrap chromatography columns (GE Healthcare, Chicago, IL, USA) with 50 mM Tris/HCl, pH 8.0. Labeled proteins were quantified by spectrophotometry and filtered with Microcon-10 kDa Centrifugal Filter Units (Millipore, Burlington, MA, USA) by spinning at 4000 rpm for 15 min in PBS. Marked proteins were quantified by UV–Vis spectroscopy and stored at 4 °C until use.

**Figure 2:**
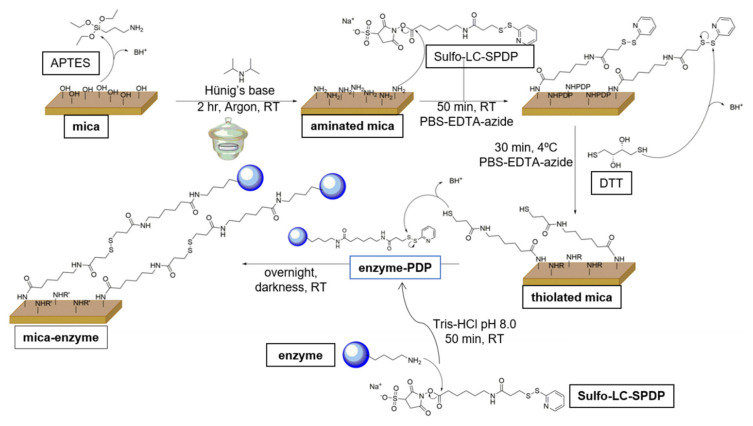
Procedure followed for the covalent immobilization of FNR on mica pieces. Mica surfaces were exfoliated with a piece of sellotape and exposed to APTES/Hünig’s base vapors in argon atmosphere to aminate the hydroxyl groups on its surface. Sulfo-LC-SDP crosslinker was then incubated with mica to transform amine groups into PDP groups, which, in the presence of DTT, were reduced to sulfhydryl reactive groups. FNR molecules were labeled with Sulfo-LC-SDP crosslinker, and then made to react with the thiolated mica to establish disulfide bonds.

Freshly cleaved 1 cm^2^ V-5 muscovite mica pieces (Electron Microscopy Sciences, Hatfield, UK) were exposed to vapors of 3-aminopropyl triethoxysilane (APTES; Sigma-Aldrich, San Luis, MO, USA) and N,N-diisopropylethylamine (Hünig’s base; Sigma-Aldrich, San Luis, MO, USA) at a 3:1 volume ratio for 2 h under an argon atmosphere (Figure 2). Then, 150 µL of 20 mM Sulfo-LC-SPDP in PBS/EDTA-azide (Thermo Scientific Pierce, Waltham, MA, USA), pH 8.3, was added to each aminated mica piece, previously fixed on 6-well ELISA plates (Thermo Scientific Nunclon, Waltham, MA, USA) with vacuum grease, and incubated for 50 min at room temperature (RT). The pieces were then washed three times in the same buffer under mild stirring to release the excess of the incubated linker molecules. The PDP groups exposed at the surfaces were reduced to sulfhydryl reactive groups by incubation with freshly prepared 150 mM dithiothreitol (DTT; Sigma-Aldrich, San Luis, MO, USA) in PBS/EDTA-azide, pH 8.3, for 30 min (Figure 2), and later washed with the same buffer under stirring [35,36].

Different amounts of the FNR-PDP samples were added on each thiolated mica piece and then incubated overnight at RT under mild stirring in darkness to form covalent disulfide bridges between them (Figure 2). The amount of enzyme-PDP was estimated to be in excess, as DFS measurements require saturated enzymatic layers to increase the specific events corresponding to the rupture of the complexes. The enzyme-functionalized mica surfaces were extensively washed under mild stirring with PBS, 0.2% Tween 20 (Panreac Química SLU, Castellar del Vallés, Spain), and 0.1% SDS (Panreac Química SLU, Castellar del Vallés, Spain), pH 8.3, to remove the loosely attached FNR molecules that might affect the AFM measurements.

### 2.2. Functionality of the Enzyme Samples

As a first control, the functionality of FNR after tagging with Sulfo-LC-SDP was ensured by evaluating its FNR cytochrome *c* reductase activity, as previously described [35]. Then, AFM topography images were obtained in a MultiMode 8 system (Bruker, Santa Barbara, CA, USA) using a soft silicon nitride 2 nm final tip radius SNL-D AFM cantilevers with a nominal spring constant of 0.06 N/m and a resonance frequency of 18 kHz in air (Microlever; Bruker Probes, Santa Barbara, CA, USA). Imaging measurements were performed using the tapping operation mode based on the cantilever oscillation near its respective resonance frequency in a liquid cell using PBS, pH 8.3. The height of the layers was analyzed by scratching experiments consisting of scraping the surface at a high loaded force in contact mode, dragging the functionalized groups throughout the surface using SNL-A probes with 0.35 N/m stiffness constant. By controlling the normal force applied to the probe, hole-type patterns can be fabricated and the mica surface can be uncovered so that a clear height profile can be obtained from an image of a larger area [37]. Analysis of the images was performed using the WxSM free software for SPM [38].

### 2.3. AFM Tip Functionalization with NADP^+^

**Figure 3:**
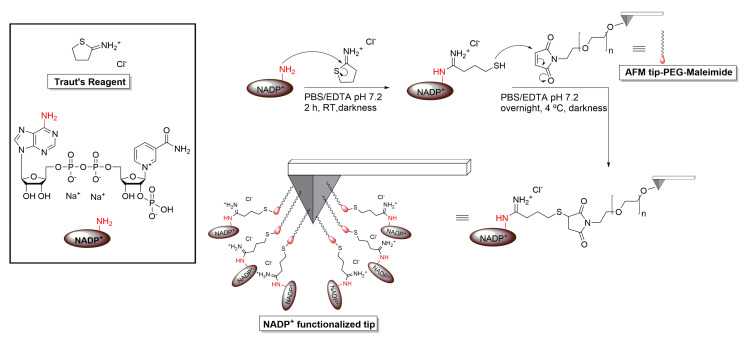
Procedure designed for the covalent attachment of NADP^+^ to AFM tips. 2-Iminothiolane reacted with the primary amine of the adenine in NADP^+^ molecules, creating amidine derivatives with reactive sulfhydryl groups. These were made to react with maleimide-PEG prefunctionalized AFM tips, producing the thiosuccinimide derivatives.

DFS requires the strong immobilization of the molecules at the cantilever probe for them not to unbind while withdrawing in force scans. Prefunctionalized maleimide-terminated flexible polyethyleneglycol (PEG) linker silicon nitride AFM cantilevers (MW 3400; Novascan Technologies Inc., Ames, IA, USA) were used. Nominal stiffness constant values of cantilevers ranged from 0.02 to 0.06 N/m. For NADP^+^ functionalization, a 2 mg/mL 2-iminethiolane solution (Traut’s Reagent; Thermo Scientific Pierce, Waltham, MA, USA) in 5 mM PBS/EDTA pH 7.2 was prepared. NADP^+^ (Sigma-Aldrich, San Luis, MO, USA) and Traut’s Reagent solution were mixed in a 1:10 molar ratio and incubated for 2 h at RT in the absence of light (Figure 3). Then, 150 μL of the aforementioned NADP^+^ mixture was added to the maleimide–PEG cantilever fixed on a capsule, and incubated overnight at 4 °C in the absence of light (Figure 3). AFM tips were washed three times (5 min each) with the same buffer and kept at 4 °C in the absence of light until use.

### 2.4. Dynamic Force Spectroscopy Measurements

AFM measurements were performed in a MultiMode 8 AFM (Bruker Digital Instruments, Santa Barbara, CA, USA) using the specialized PicoForce scanner (Bruker, Santa Barbara, CA, USA). Several hundred force–distance (Fz) cycles were registered for NADP^+^-tip/FNR-mica from different sample locations at each different loading rate (R) [39]. Fz curves were collected varying the velocity of 50–4000 nm/s. The loaded force between the functionalized tip and the sample was kept constant at 1.25 nN. These data translate into R, varying from 3 to 80 nN/s. The Fz curves were collected as voltage versus distance scans [39]. In order to convert the voltage into force units, the spring constant of the functionalized cantilever and the deflection sensitivity (inverse of the optical lever sensitivity, in nm/V) were used [39]. Measurements were performed inside a liquid cell in PBS/EDTA, pH 7.2 at RT. In control experiments, samples were incubated with 2.6 mM NADP^+^ in PBS/EDTA-azide, pH 8.3, for 15 min at R 10 nN/s in order to block the available FNR sites.

### 2.5. Analysis of the Force Curves: Mechanostability Parameters

**Figure 4:**
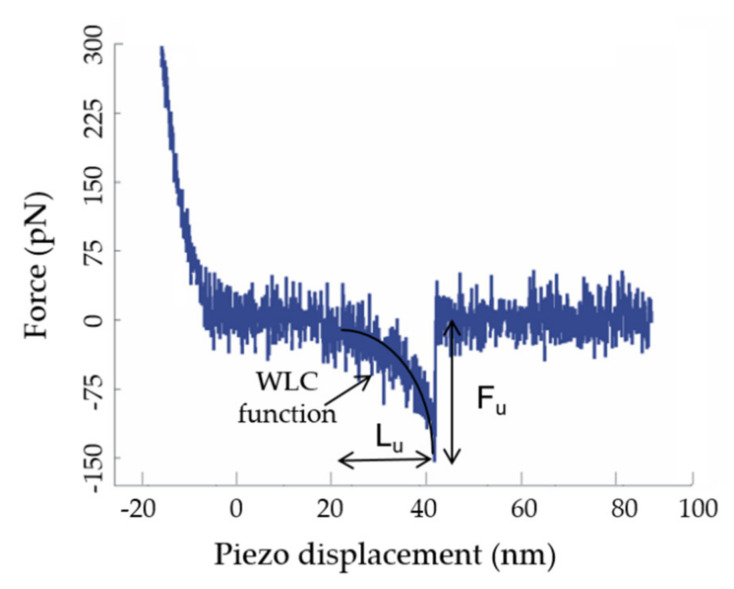
Representative experimental retraction force curve showing a specific unbinding event corresponding to the rupture of a single AnFNR_ox_:NADP^+^ complex. Starting from the zero-force point, the tip is moved closer to the sample with an increment in the force until tip and sample come into contact (approach curve not shown for picture clarity. See [36]). Pushing the tip further towards the surface requires higher forces, causing the bending of the cantilever. If a bond is formed during approach, a sharp jump (the peak seen in the curve at 40 nm) is produced during retraction of the tip (blue line), indicating that a sudden release has occurred between tip and sample. To attribute this force peak (F_u_) to a specific unbinding event, the rupture of the bond between the two interacting molecules should present an unbinding length (L_u_) or tip–sample separation close to the length of the stretched linker, around 20 nm, given by the piezo displacement encompassing the non-linear portion of the retraction curve before unbinding. The black continuous curved line represents the corresponding PEG stretch according to the WLC function [40]. The shape of the force peak and the distance at which it occurs ensure that measured forces come from recognition events and not from artifacts or non-specific tip–sample adhesion.

Statistical analysis is mandatory, since complex formation is a stochastic process. Histograms of the frequency of the forces versus the corresponding rupture forces of the specific unbinding events were built for each loading rate. Peak force data were only processed from Fz curves when they met two specificity requirements: (i) Fz curves were produced at a distance coinciding with the length of the stretched PEG spacer that binds the NADP^+^ molecule to the tip; and (ii) their shape coincides with the stretching function of the corresponding PEG molecule [39,40] (Figure 4). The value for the stretched PEG spacer used in this work is around 20 nm [41]. 

Histograms unbinding force distributions were fitted with Gaussian functions without shifting or using zero-truncated strategies. Fitted maxima with lower values were assigned to the most probable unbinding force for a single rupture event (F*) at the measured R. Finally, the representation of the most probable unbinding forces versus the logarithm of R following the Evans–Ritchie expression (1) [42] allowed us to estimate the dissociation rate constant at zero force, *k_off_*, and the distance of the energy barrier with respect to the ordinate axis, *x_β_*, to characterize the mechanostability of the complexes and the number of transitions in the process.

(1) $$F^{*}=\left(\frac{k_{B}\;T}{x_{\beta }}\right)\ln\left(\frac{R\;x_{\beta }}{k_{\mathrm{off}}\;k_{B}\;T}\right)$$

## 3. Results

### 3.1. Analysis of the Enzymatic Samples

The study of the dissociation of biomolecular complexes by DFS requires the strong attachment of both biomolecules of interest: one to the AFM substrate and the other to the AFM cantilever tip. This requires molecules to be labelled in advance while preserving their catalytic function. The UV–Vis absorption spectra of FNR-PDP solutions displayed all the UV–Vis absorption features of the unmarked enzymes, showing the flavin band-II maxima at 459, 456, 460, and 456 nm for WT, Y303S, Y303F, and Y303W, respectively (not shown), indicating that their overall conformations were not affected significantly by the labeling. In addition, the labeled WT-FNR retained ~70% of its activity under steady-state conditions. Thus, the labeling procedure has no major effect on either the FNR conformations or on their catalytic activity. 

**Figure 5:**
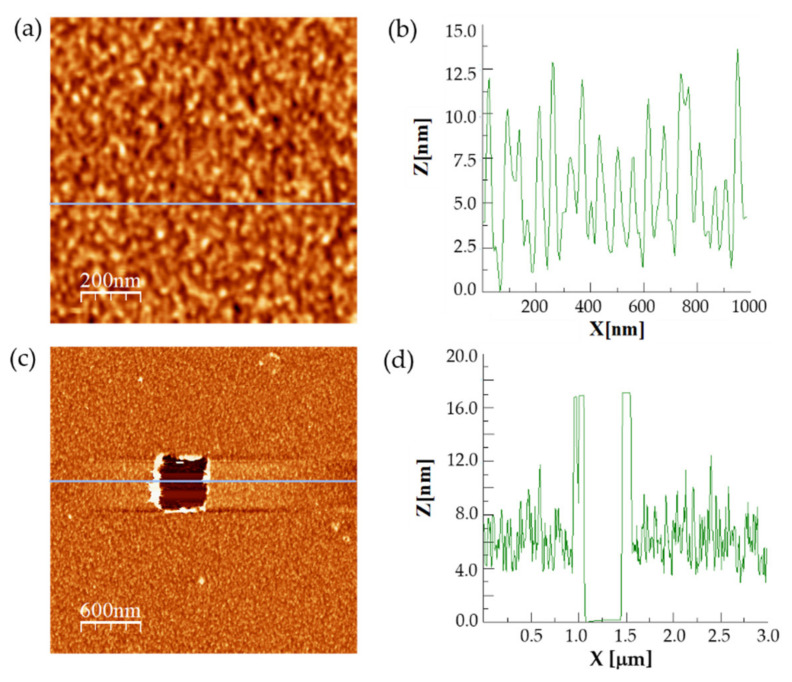
Analysis of the enzymatic samples through AFM. (**a**) Representative AFM topography image of a sub-monolayer Y303S sample used to perform the DFS measurements. (**b**) Height profile of the corresponding blue line in (**a**). (**c**) Topography image of a Y303S sample after scratching an area of 500 × 500 nm. (**d**) Height profile of the corresponding blue line on the center on the scratched area in (**c**), exhibiting a hole corresponding to a monolayer of FNR.

AFM imaging was also used as a tool to further monitor FNR samples at the nanometer level after successive chemical modification steps. Different amounts of FNR solutions were incubated on the thiolated mica pieces (Figure 2). A minimum of 4 µg of FNR per mica piece was required to fully cover the functionalized mica surfaces (not shown), so higher amounts were added. Detergent treatment was shown to efficiently avoid the protein aggregation effects that might detrimentally affect the DFS measurements. The height of the protein monolayers was precisely determined from the cross-sectional profiles. The average height in all the samples was 8 nm (Figure 5a,b). Scratching experiments were performed in addition to confirm that the thickness of the protein layers was the same (Figure 5c,d). These data were in accordance with the molecular size, of around 2.9 × 5.4 × 5.7 nm in 3D, for WT FNR (PDB 1que) and Y303S (PDB 2bsa), and the different chemical functionalization steps, estimated as 0.6 nm for APTES-modified mica and 1.2 nm for thiolated-modified mica [35]. Thus, they correspond to the sub-monolayers of enzyme molecules in all the immobilized samples.

### 3.2. Mechanical Stability of the FNR Complexes

**Figure 6:**
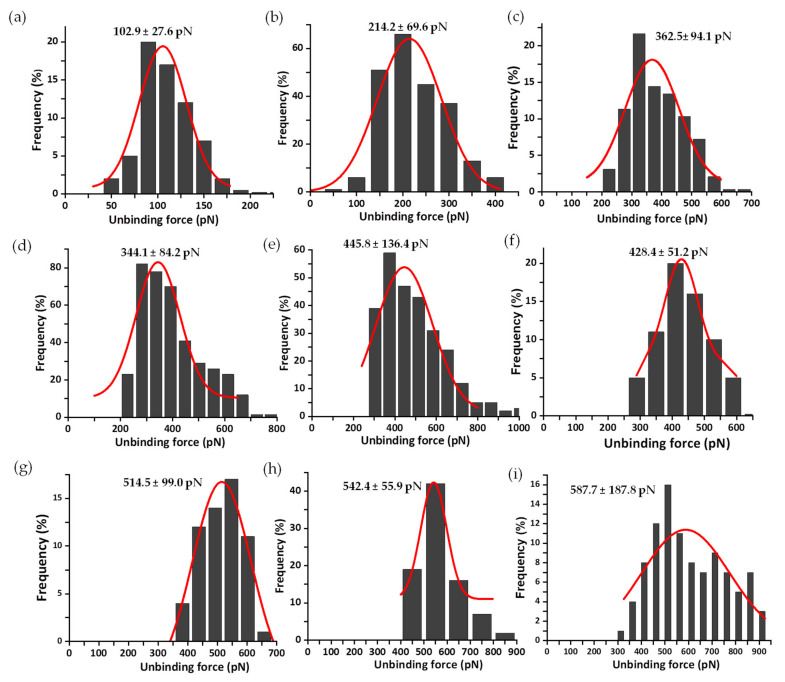
Force histogram distributions for WT AnFNR_ox_:NADP^+^ complexes. Data force obtained operating at R values of 6 (**a**), 13 (**b**), 20 (**c**), 25 (**d**), 30 (**e**), 40 (**f**), 60 (**g**), 65 (**h**), and 78 (**i**) nN/s. The width of the bars varies in each case, depending on the optimum fit, with the Gaussian function shown in red. Data from R 10 nN/s were also measured, but are shown below. Each fitting provides the most probable unbinding force corresponding to the rupture of a single complex and is shown on the corresponding graphic.

Thousands of Fz curves were collected for the WT AnFNR_ox_:NADP^+^ complexes, as well as for those of its C-terminal Y303 variants at several R values. Their analysis was consistently performed in order to discard all those force peaks that do not reflect specific interactions or ambiguous ones that are technically considered as “false events”. The percentage of specific events ranged from 5 to 18% with respect to the total attempts for all samples, despite using saturated samples to enhance encounter and bond probabilities between both molecules. These values are common for this type of experiment using random labelling procedures. Thus, only peak force data that fit the requirements described in Section 2.4 and Figure 4 were considered for the composition of force histograms at different R values [39]. Figure 6 summarizes the histograms obtained for the unbinding forces of WT AnFNR_ox_:NADP^+^ complexes at several R values. The most probable unbinding forces estimated for WT AnFNR_ox_:NADP^+^ range from 103 ± 27 to 588 ± 188 pN at R values from 6 to 78 nN/s (Figure 6).

**Figure 7:**
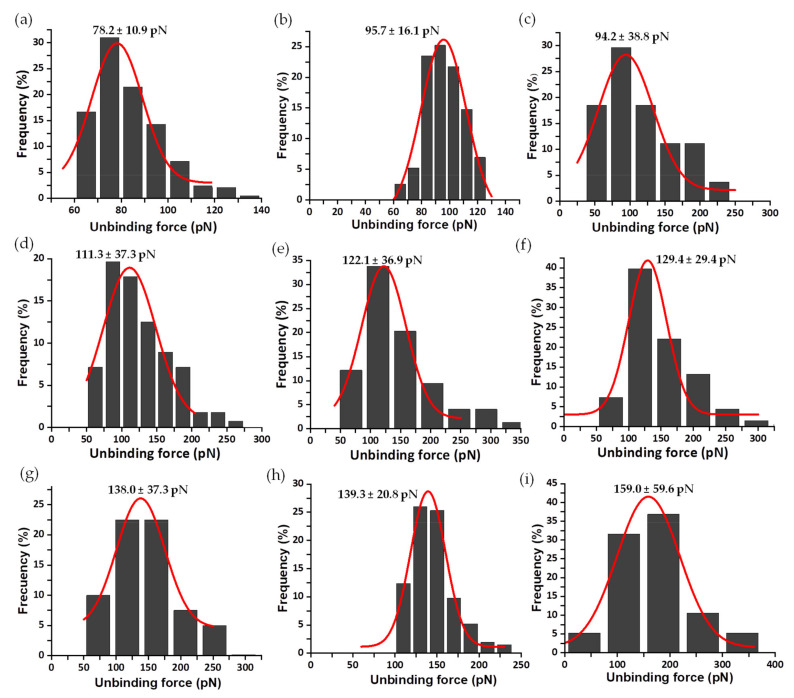
Force histogram distributions for Y303S AnFNR_ox_:NADP^+^ complexes. Data force obtained operating at R values of 3 (**a**), 20 (**b**), 31 (**c**), 43 (**d**), 50 (**e**), 61 (**f**), 69 (**g**), 79 (**h**), and 88 (**i**) nN/s. The width of the bars varies in each case, depending on the optimum fit with the Gaussian function shown in red. Data from R 10 nN/s were also measured, but are shown below. The fitting provides the most probable unbinding forces corresponding to the rupture of a single complex and is shown on the corresponding graphic.

**Figure 8:**
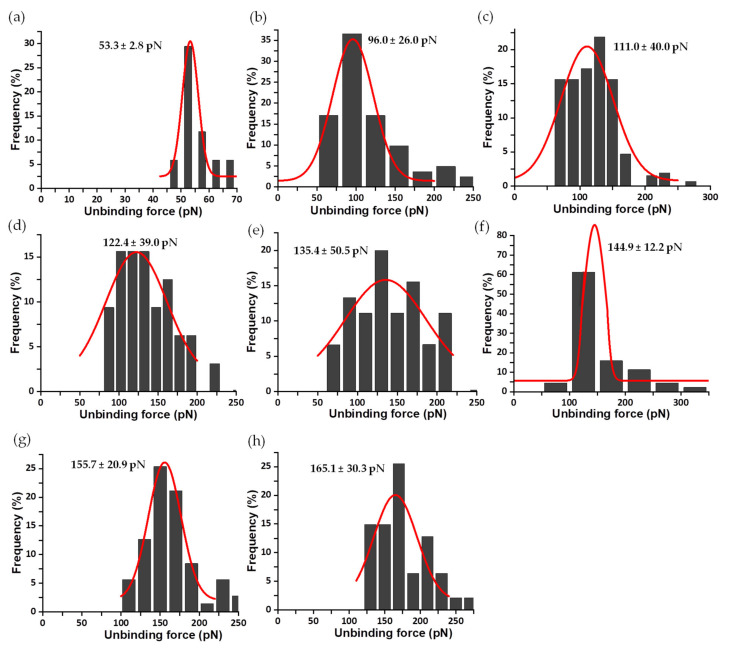
Force histogram distributions for Y303F AnFNR_ox_:NADP^+^ complexes. Data force obtained operating at R values of 3 (**a**), 21 (**b**), 28 (**c**), 46 (**d**), 57 (**e**), 65 (**f**), 77 (**g**) and 96 (**h**) nN/s. The width of the bars varies in each case, depending on the optimum fit with the Gaussian function shown in red. Data from R 10 nN/s were also measured but are shown below. The fitting provides the most probable unbinding forces corresponding to the rupture of a single complex and is shown on the corresponding graphic.

**Figure 9:**
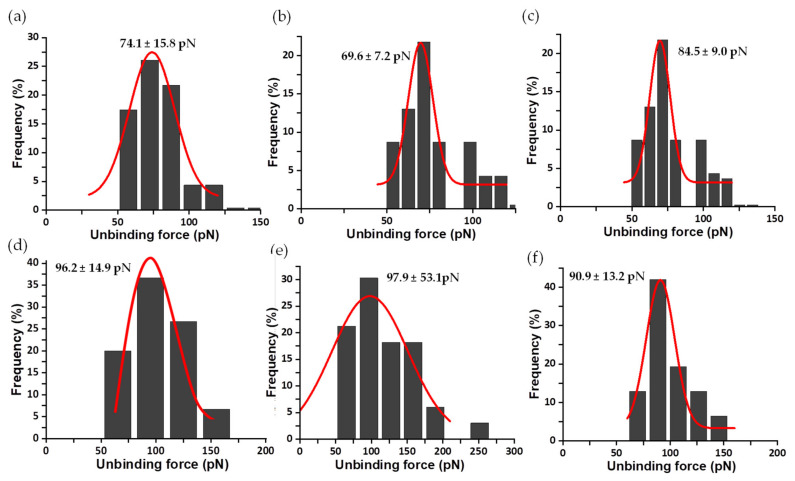
Force histogram distributions for Y303W AnFNR_ox_:NADP^+^ complexes. Data force obtained operating at R values of 20 (**a**), 31 (**b**), 40 (**c**), 52 (**d**), 58 (**e**), and 75 (**f**) nN/s. The width of the bars varies in each case, depending on the optimum fit with the Gaussian function shown in red. Data from R 10 nN/s were also measured, but are shown below. The fitting provides the most probable unbinding forces corresponding to the rupture of a single complex and is shown on the corresponding graphic.

Sometimes, as seen with the analysis of FNR complexes with its protein partners, Fd and Fld [33], each asymmetrical force histogram might be grouped under one or more different peaks fitted to Gaussian curves. However, in this study of the interaction of FNR with the coenzyme, it was rare to find rupture events corresponding to multiple complexes. Therefore, for clarity, only those attributed to individual complexes are evaluated here. The same methodology was used to analyze the mechanostability of the three complexes formed by the coenzyme and the C-terminal Y303 FNR variants. The mean value of unbinding forces ranges from 78 ± 11 to 159 ± 60 pN for Y303S FNR_ox_:NADP^+^ at R from 3 to 88 nN/s (Figure 7); 53 ± 3 to 165 ± 30 pN for Y303F FNR_ox_:NADP^+^ at R from 3 to 96 nN/s (Figure 8); and 68 ± 15 to 91 ± 13 pN for Y303W FNR_ox_:NADP^+^ at R from 10 to 78 nN/s (Figure 9). The gathered data estimated at 10 nN/s indicate that the complex WT AnFNR_ox_:NADP^+^ is 1.6, 1.8, and 2.0 times stronger than Y303S AnFNR_ox_:NADP^+^, Y303F AnFNR_ox_:NADP^+^, and Y303W AnFNR_ox_:NADP^+^, respectively. The frequency of successful events of the latter was particularly low for all conditions, suggesting that the tryptophan residue in that position does not favor recognition of the coenzyme.

**Figure 10:**
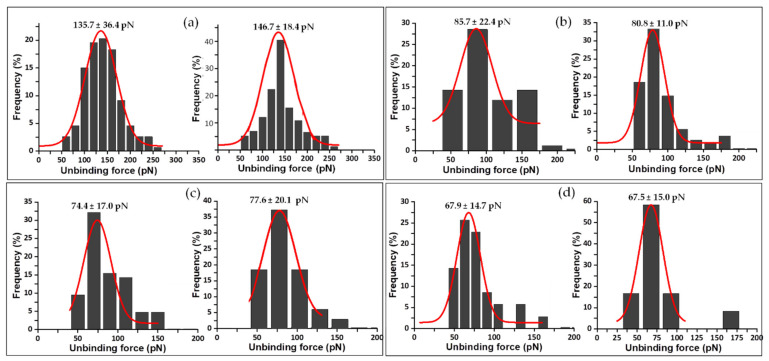
Control experiments showing histograms obtained at R 10 nN/s for AnFNR_ox_:NADP^+^ complexes and the corresponding blocked samples with excess ligand. Unbinding force data obtained for (**a**) WT AnFNR_ox_:NADP^+^, (**b**) Y303S AnFNR_ox_:NADP^+^, (**c**) Y303F AnFNR_ox_:NADP^+^, and (**d**) Y303W AnFNR_ox_:NADP^+^. Data obtained from blocked samples appear to the right in the corresponding panel. The width of the bars varies in each case, depending on the optimum fit with the Gaussian function shown in red. The fitting provides the most probable unbinding forces corresponding to the rupture of a single complex and is shown on the corresponding graphic.

Force data acquired at R 10 nN/s are commonly used to compare the estimated unbinding forces across different systems (Figure 10, Table 1). The specificity of the measurements was also evaluated by blocking experiments at this loading rate (Figure 10). The probability of complex formation is reduced by the binding of free ligand to the same enzyme binding site. The fitting of the resulting histograms has very similar maximal data to those from non-blocked samples at the same conditions, but shows a decreasing event frequency. This is not visually evident in the histograms as they only show the relative frequency between specific forces, without including the non-specific ones. However, the frequency of the rupture events decreased 3-fold—from 12 to 4% (non-blocked and blocked samples, respectively)—highlighting the specificity of the intermolecular interaction forces measured. The results obtained from the blocking experiments are clearly indicative of the specificity of the measurements, as can be seen in Figure 10. The calculated most probable rupture forces for non-blocked/blocked samples are 136 ± 36/147 ± 18; 86 ± 22/81 ± 11; 74 ± 17/77 ± 20 and 68 ± 15/67 ± 15 pN for WT AnFNR_ox_:NADP^+^, Y303S AnFNR_ox_:NADP^+^, Y303F AnFNR_ox_:NADP^+^, and Y303W AnFNR_ox_:NADP^+^, respectively.

### 3.3. Dissociation Kinetics for AnFNR_ox_:NADP^+^ Complexes

A typical energy landscape is a one-dimensional plot that represents the energy of the system versus the reaction coordinate. In the case of dissociation under force, a force-driven pathway along the pulling direction upon tip retraction defines the bond rupture [10,43]. The shape of this particular landscape is defined by the height of the energy barrier, characterized by the dissociation rate constant at zero force, *k_off_*, and the energy barrier width between the valley and the summit of the peak, the *x_β_* parameter. 

**Figure 11:**
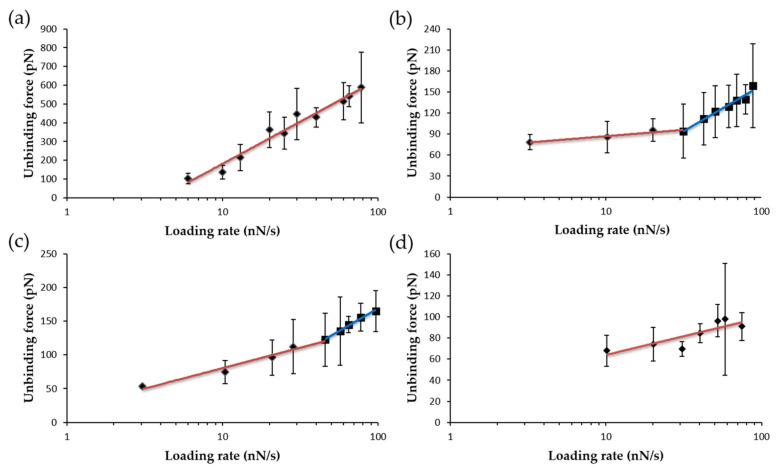
Loading-rate dependence of the most probable unbinding forces. Unbinding forces corresponding to the rupture of single complexes that result from the Gaussian fit to the histogram distributions of WT FNR_ox_:NADP^+^ (**a**), Y303S FNR_ox_:NADP^+^ (**b**), Y303F FNR_ox_:NADP^+^ (**c**), and Y303W FNR_ox_:NADP^+^ (**d**). Force statistical errors are given by standard deviation. The solid lines correspond to numerical fit of experimental data to the Evans–Ritchie expression [42]. Data from WT and Y303W FNR_ox_:NADP^+^ complexes optimally fit to one straight line (red), whereas data from Y303S and Y303F FNR_ox_:NADP^+^ complexes optimally fit to two straight lines (red and blue). Best-fitting nanomechanical parameters are shown in Table 1.

Representation of the most probable unbinding forces (F*) versus the logarithm of R for the WT FNR_ox_:NADP^+^ complex indicated a linear dependence (Figure 11a). Such behavior can be traced back to a single-step dissociation process of NADP^+^ from the FNR:NADP^+^ complex. Conversely, the same representation gives two linear regimes for complexes formed by Y303S and Y303F mutants with NADP^+^ (Figure 11b,c). Such behavior is attributed to the presence of two events, with the appearance of one intermediate state in the dissociation process. This suggests that NADP^+^ molecules dissociate from FNR through two energy barriers [44]. Finally, force data from the dissociation of the Y303W FNR_ox_:NADP^+^ complex was a better fit to a single straight line (Figure 11d). When two events or loading rate regimes are observed, the inner energy barrier is expected to be crossed first and then followed by the crossing of a second outer energy barrier [44]. In addition, it is accepted that the application of a linear force on a bond causes the outer energy barrier of the energy landscape to decrease. Consequently, when present, the inner energy barrier can be observed by applying higher R [42].

Table 1 summarizes the main parameters obtained from fittings of the graphics in Figure 11, including the half-life, *τ*(s), which is the inverse of *k_off_* [45]. The *k_off_* value for the single detected event in the dissociation of NADP^+^ from WT FNR_ox_ indicates a considerably slower process than the previously reported dissociation of complexes of the enzyme with its protein partners, Fd and Fld [33,34]. Dissociation of the coenzyme from the Y303W mutant, also characterized by a single dissociation event, occurs with a *k_off_* that decreases by only two-fold compared to the WT. Dissociation of the coenzyme from complexes of Y303S and Y303F variants occurs in two steps and these processes are therefore characterized by two *k_off_* values. Notably, these values differ from the two steps within a particular mutant, as well as among both mutants (Table 1).

Thus, *k_off_* values for the dissociation of NADP^+^ from the Y303S FNR complex indicate that while one of the events occurs 5 times faster than the single event observed for the WT complex, the other is up to 1000 times slower. However, in the case of the Y303F complex, one of the processes is in the same range as in for the WT complex, and the other is 5 times faster. These parameters show that the WT complex has the shortest half-life, followed closely by the Y303F and Y303W complexes, whereas one of the two dissociation events for the Y303S complex lasts so long that it hardly occurs (Table 1). It is worth noting that all mutations, particularly Y303S, produce considerably higher ***x_β_*** values. This suggests the need for an increase in the distance among dissociation elements when Y303 is substituted by Phe, Trp and, particularly, Ser.

## 4. Discussion

The great biological significance of enzyme:substrate interactions contrasts with the absence of nanomechanical data for these complexes that, to the best of our knowledge, exists to date. Fz scans provide reliable data about the interaction and dissociation processes and represent the large variability associated with their stochastic nature (random or non-deterministic processes).

The mechanical parameters estimated by DFS demonstrated the strong and specific interactions that governs the physiological WT AnFNR_ox_:NADP^+^ complex: (i) a high most-probable unbinding force, 135.7 ± 36.4 pN at R 10 nN/s; (ii) a single event in the dissociation process; and (iii) a stable complex, *k_off_* 0.0202 s^−1^, and τ 49.42 s. The WT AnFNR_ox_:NADP^+^ complex is mechanically stronger than most of the measured biological systems reported so far. The three enzyme variants decrease the mechanical strength of their corresponding complexes to a similar extent, to approximately half the value of the WT, highlighting the relevance of the Tyr residue at C-terminus for the formation of a stable complex of the enzyme with NADP^+^.

The mechanostability of the WT AnFNR_ox_:NADP^+^ complex is remarkably higher (by almost 3.5 and 6.5 times) than that estimated for the complexes of AnFNR with its protein redox partners [33,34]. This agrees with data accumulated over the years showing that the interaction of FNR with the coenzyme is stronger and more specific than with these proteins [18,19,20]. Even the forces found for the three variants are higher than those for protein complexes (Table 1). These observations can be explained since FNR binds specifically to NADP^+^—its affinity for NAD^+^ is negligible [22]—but exchanges efficiently electrons with either Fd or Fld, two dissimilar proteins that bind to FNR at the same site. Close to the mechanical strength found for the WT AnFNR_ox_:NADP^+^ complex, we find azurin:cytochrome c551 [46] (140 pN) and p53:Mdm2 (130 pN) complexes [47] (at R 10 nN/s).

One striking finding in our study is the high value of the dissociation rate of the WT FNR_ox_:NADP^+^ complex, 0.0202 s^−1^, which is among published typical values of cell adhesion complexes, around 0.1 s^−1^, and antigen:antibody pairs, around 0.001 s^−1^. These values are far from those reported for transient complexes, typically between 10–1000 s^−1^, as is the case for the FNR:Fd/Fld redox pairs [33]. The constant *k_off_*, related to the height of the energy barrier, strongly depends on the interaction properties of the partners. Similar *k_off_* values were estimated for strong pairs such as P-selectin:P-selectin ligand (0.022 s^−1^) [48]; anti-digoxigenin:digoxigenin (0.015 s^−1^) [49]; p53:azurin (0.09 s^−1^) [50]; and erythrocyte:fibrinogen complexes (0.025 s^−1^) [51]. FNR variants that retain an aromatic residue at the 303 C-terminal position, Y303F and Y303W, did not change *k_off_* significantly compared to the WT value (Table 1). In the case of Y303S, one of the *k_off_* values decreased drastically, to 0.00002 s^−1^, forming a complex as stable as the streptavidin:biotin system, 0.000017 s^−1^ [44], one of the strongest known non-covalent protein:ligand complexes in nature (measured to date).

Moreover, the Bell–Evans interpretation of the energy landscape [42] may provide useful information related to the chemistry of binding. For instance, a small energy barrier width, *x_β_* ≤ 0.1 nm, likely involves the rupture of hydrogen bonds or salt bridges from a rigid ligand [52], while a high *x_β_*, ≥1 nm, presumably implies a deformation of one or both partners before the rupture. According to this, the value of *x_β_* for the WT AnFNR_ox_:NADP^+^ complex, 0.021 nm, suggests a small deformation in the dissociation or just the breaking of some bonds, that gets larger in all the variants: first Y303F, second Y303W, and finally, Y303S FNR. This indicates the biggest conformational change before dissociation—0.524 nm—of one or both partner molecules and it is similar to those reported for FNR protein complexes [33].

**Figure 12:**
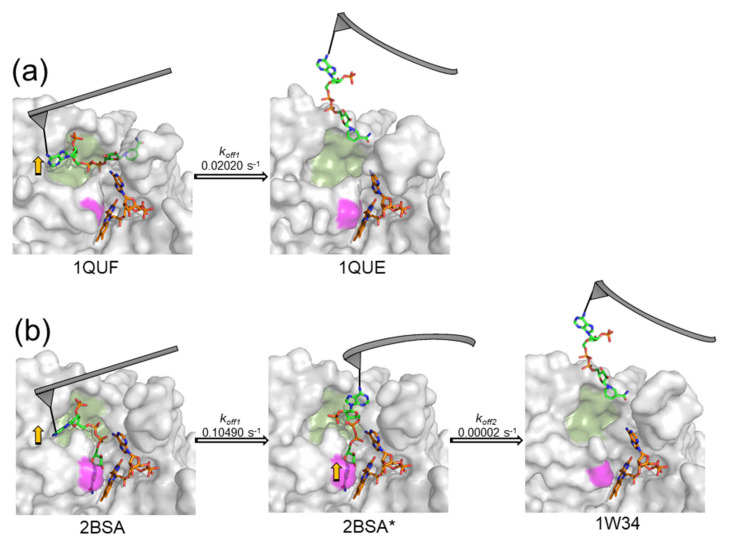
Scheme of force dissociation kinetics steps for (**a**) WT AnFNR_ox_:NADP^+^ and (**b**) S303 AnFNR_ox_:NADP^+^. NADP^+^ is covalently bound to the AFM tip (grey color) through the primary amine of its adenine; therefore, the flexible AFM cantilever pulls from the adenine to dissociate the coenzyme from the enzyme. The FNR enzyme bipartite binding sites for NADP^+^ are coloured in green (2′P-AMP site (1)) and in pink (nicotinamide site (2)). The *k_off_* values related to each dissociation step are depicted above the arrows. Yellow arrows indicate where pulling forces are proposed to cause dissociation at each step. Protein structures are based on crystallographic structures with the corresponding PDB codes underneath, except 2BSA* where the intermediate state with NADP^+^ dissociated only from the 2′P-AMP site has been modeled.

In WT AnFNR, the nicotinamide is only expected to gain access to the active site to couple with the FAD isoalloxazine when flavin reduction has occurred by accepting electrons from the protein partner, thus enabling transient competent binding for catalysis [18,19,20]. Therefore, the determined parameters for the single dissociation event detected for the WT FNR_ox_:NADP^+^ complex must correspond to C-I and/or C-II conformations in Figure 1a, where coenzyme binding is mainly attributable to the 2′-P-AMP moiety. The Tyr residue at the C-terminus of plastidic WT FNRs forms a parallel π–π stacking interaction with the FAD isoalloxazine, preventing access of the coenzyme nicotinamide to the active site, and is key to maintaining a high enzyme turnover by contributing to the coenzyme release from the active site [23,24,29,30,53]. The removal and substitution of the Tyr side-chain for Ser considerably increases the enzyme affinity for NADP^+^ by particularly favoring nicotinamide allocation at the enzyme active site [23,24,29]. In such mutants, the strong stacking between the flavin isoalloxazine and the nicotinamide coenzyme has been proposed to lead the interaction (Figure 1b), as shown by the breaking in selectivity for NADP^+^ versus NAD^+^ and the full occupancy of the active site by the nicotinamide [29]. Thus, the two-event process observed here when plotting the most probable unbinding forces versus R for the NADP^+^ dissociation from Y303S FNR (Figure 11b) can be assigned to the dissociation of the 2′-P-AMP and nicotinamide moieties from their corresponding enzyme bipartite binding sites (Figure 12). Therefore, these plots allow us to determine for the first time the mechanical parameters at each of these sites, which is very different at the bipartite sites of Y303S. This could be similar to the proposed existence of two binding sites associated to two well-distinct linear regimes for the interaction between holo-transferrin and its receptor and a single site for the apo-form that corresponds to a single loading rate regime [54].

Considering that NADP^+^ is anchored to the AFM tip through the adenine of its 2′-P-ADP nucleotide, we can predict that the dissociation of its nicotinamide moiety is the event exhibiting the largest *x_β_* value and, therefore, the lowest *k*_off_ (Figure 12). This agrees with studies confirming that Y303S FNR favors the strong stacking of the coenzyme nicotinamide to the FAD isoalloxazine at the active site [26]. Such a strong coupling has a negative impact on the enzyme turnover [24,29]. Thus, in the WT, displacement of Y303 has to occur for the catalytically isoalloxazine–nicotinamide competent orientation to be achieved, but Y303 remains at the active site to reduce the stacking probability between the reacting rings that takes place in the Y303S FNR:NADP^+^ complex [30,53]. Parameters for the other event indicate a shorter *x_β_* and a faster *k*_off_, and should therefore correspond to the dissociation of the 2′-P-AMP moiety. Intermediate behaviors between WT and Y303S were observed for NADP^+^ dissociation from the two remaining mutants. Y303W exhibited a single event for the NADP^+^ dissociation as in WT FNR, whereas stabilization of one intermediate characterizes the dissociation from Y303F. This agrees with previous studies showing that the estimated occupancy of the NADP^+^ nicotinamide at the FNR_ox_ active site is up to 100% and 71% for Y303S and Y303F, respectively, while these values drop to 15% and 0% for Y303W and WT [29]. Moreover, the parameters related to the dissociation of either the nicotinamide or 2′-P-AMP moieties differ among variants. This observation is not strange, since coupling of the nicotinamide to the active site also influences the conformation of the 2′-P-AMP and pyrophosphate moieties and of their corresponding binding cavities (Figure 1) [27].

Beyond the specific information regarding the dissociation of AnFNR_ox_:NADP^+^ complexes and the role of Y303 in blocking nicotinamide access to the access site, this study paves the way to use DFS by AFM to characterize, at the single-molecule level, the occurrence and relevance of bipartite binding sites in pyridine nucleotide-dependent enzymes. In line with this, by considering plastidic and bacterial FNRs, we can envision different profiles among family members with different adaptation of their active sites to divergent dynamics and organizations during catalysis, with recognition mechanisms that either favor the initial binding of either of the two nucleotide moieties of the coenzyme [55,56]. To complete the study of the interactions in this electron transfer chain, it would be desirable to operate DFS also by preserving the FAD cofactor and the NADP^+^ substrate, in the oxidized and in the reduced state, respectively. This will require complex technical developments enabling AFM measurements while maintaining only one of these molecules in the reduced state, which is not currently available.

## 5. Conclusions

The presented data provide a comprehensive dissection of the FNR:NADP^+^ dissociation process. They lead to novel conclusions about the kinetics and energetics of the binding of the NADP^+^ substrate to FNR, and highlight the bipartite coenzyme binding/dissociation mode and the role of the enzyme C-terminal Tyr residue in the process. In addition, the study further shows DFS by AFM is a powerful technique for providing new insights and unraveling the molecular mechanisms underlying the formation and dissociation of enzyme:coenzyme or enzyme:ligand systems. As far as we know, it is remarkable that the present work tackles the first enzyme:substrate system analyzed by DFS at the single-molecule level. Therefore, not only does it contribute to better understanding of this particular FNR system, but it also opens the door to the analysis of other enzyme:ligand systems. Additionally, the method designed here to immobilize NADP^+^ can be used for DFS, as well as for any other approach that requires it to be anchored to a surface. The acquired knowledge is not only interesting from the molecular point of view but can also be useful to design artificial enzymatic systems offering enhanced catalytic performances for the production of useful molecules or with higher sensitivity for bio-sensing applications.

## Figures and Tables

**Table 1:** Mechanical parameters for the dissociation of FNR_ox_:NADP^+^ complexes. The values were obtained from fitting of data shown in Figure 11. Values for the AnFNR_ox_:Fd_ox_/Fld_ox_ complexes were previously reported [33] and added here to facilitate discussion. Assays were performed in PBS/EDTA pH 7.2.

Complex	Unbinding Force (pN) *	Events	*k_off_* (s^−1^)	*τ* (s)	*x_β_* (nm)
WT FNR_ox_:NADP^+^	135.7 ± 36.4	1 barrier	0.02020	49.42	0.021
Y303S FNR_ox_:NADP^+^	85.7 ± 22.4	inner barrier outer barrier	0.10490	9.53	0.073
			0.00002	50,838.84	0.524
Y303F FNR_ox_:NADP^+^	74.4 ± 17.0	inner barrier outer barrier	0.01810	56.13	0.157
			0.09410	10.61	0.071
Y303W FNR_ox_:NADP^+^	68.0 ± 14.7	1 barrier	0.01020	98.29	0.267
WT FNR_ox_:Fd_ox_	57 ± 16	1 barrier	21.2	0.047	0.27
WT FNR_ox_:Fld_ox_	21 ± 8	inner barrier	253.3	0.004	0.18
		outer barrier	55.7	0.018	0.47

* Values corresponding to the rupture of a single complex, at R 10 nN/s.

## Data Availability

Data contained within the article.
